# Accelerated Long-Term Hearing Loss Progression After Recovery From Idiopathic Sudden Sensorineural Hearing Loss

**DOI:** 10.3389/fneur.2021.738942

**Published:** 2021-12-08

**Authors:** Samuel Early, Jens C. van der Valk, Johan H. M. Frijns, Konstantina M. Stankovic

**Affiliations:** ^1^Eaton-Peabody Laboratories, Department of Otolaryngology—Head and Neck Surgery, Massachusetts Eye and Ear, Boston, MA, United States; ^2^Department of Otolaryngology—Head and Neck Surgery, Harvard Medical School, Boston, MA, United States; ^3^Division of Otolaryngology—Head and Neck Surgery, Department of Surgery, University of California, San Diego Medical Center, San Diego, CA, United States; ^4^Leiden University Medical Center, Leiden, Netherlands; ^5^Department of Otolaryngology, Head and Neck Surgery, Leiden University Medical Center, Leiden, Netherlands; ^6^Leiden Institute for Brain and Cognition, Leiden, Netherlands; ^7^Department of Otolaryngology Head and Neck Surgery, Stanford University School of Medicine, Stanford, CA, United States

**Keywords:** sudden hearing loss, recovery, progression, ipsilateral, contralateral

## Abstract

**Background and Introduction:** Idiopathic sudden sensorineural hearing loss (ISSNHL) is characterized by rapid onset, typically unilateral presentation, and variable recovery. This case-control observational study aimed to improve patient counseling by objectively characterizing long-term hearing loss progression following ISSNHL, using sequential audiometry in the largest-to-date cohort of patients with ISSNHL.

**Methods:** Patients diagnosed with ISSNHL at a tertiary referral hospital from 1994 through 2018 with sequential audiometry were studied. Case controls with sensorineural hearing loss (SNHL) were matched by age, sex, baseline hearing status, and frequency of sequential audiometry. Hearing loss progression was quantified using Kaplan–Meier (K–M) analysis to account for variable follow-up duration. A subgroup analysis was performed by age, sex, preexisting comorbidities, ISSNHL-associated symptoms, ISSNHL treatment, and degree of post-ISSNHL hearing recovery.

**Results:** A total of 660 patients were identified with ISSNHL. In patients with post-ISSNHL recovery to good hearing [pure tone average (PTA) <30 dB and word recognition score (WRS) > 70%], median time to progression to non-serviceable (PTA > 50 dB or WRS <50%) SNHL was 16.4 years. In patients with incomplete post-ISSNHL hearing recovery, contralateral ears were also at significantly higher risk of SNHL progression over the following 12-year period. Male sex was associated with increased risk of SNHL progression [odds ratio (*OR*) 3.45 male vs. female] at 5-year follow up. No other subgroup factors influenced the likelihood of SNHL progression.

**Discussion and Conclusion:** Patients should be counseled on continued risk to long-term hearing after stabilization of hearing post-ISSNHL, with particular emphasis on greater risk to the contralateral ear in those with incomplete ipsilateral recovery.

## Introduction

Idiopathic sudden sensorineural hearing loss (ISSNHL) occurs most often without any obvious triggering cause, reaches maximum deficit within an acute time period, and demonstrates variable recovery (1). According to the most recent guidelines from the American Academy of Otolaryngology—Head and Neck Surgery (AAO-HNS), ISSNHL is defined by ≥30 dB hearing loss across three consecutive frequencies that cannot be attributed to an underlying condition such as tumor, stroke, noise exposure, or ototoxic medication (2). Nationwide incidence of ISSNHL is estimated at 27 per 100,000 annually (3).

The etiology for ISSNHL is poorly understood and likely multifactorial. Previous research has supported a wide range of underlying comorbidities as being positively associated with ISSNHL, such as vascular, metabolic, allergic, autoimmune, and depressive disorders (4–11). Environmental factors such as weather patterns have been explored as a potential contributor to disease incidence with little in the way of significant findings, with the possible role of seasonally-associated factors being alternately supported or refuted (12–16). Current standard of care for treatment involves either oral or intratympanic glucocorticoids; other treatments, such as hyperbaric oxygen therapy or antiretroviral therapies are commonly used, although without evidence that they modify disease progression (2).

The studies to date that have looked into recovery after ISSNHL are generally single-center, retrospective analyses performed on small patient populations (17, 18). While these studies have typically included robust audiometric data in their analyses, small patient population sizes have limited the degree to which an analysis can be performed on sub-populations or the role of patient comorbidities in ISSNHL prognosis. Larger studies, on the other hand, rely only on diagnostic or billing codes for identification of patients with ISSNHL, and are limited by lack of audiometric data to fully characterize severity and evolution of hearing loss over time (14, 19–21). A nationwide multicenter observational study in Japan showed various factors associated with severity of hearing impairment and prognosis, however with emphasis primarily on patients with a poor prognosis after initial ISSNHL, and without evaluation of hearing loss progression over time (22).

Nowhere in the current literature exists a study of patients with ISSNHL that combines both a large enough patient population and comprehensive audiometric data to be capable of definitively characterizing patient demographics and comorbidities that influence the hearing loss progression after recovery form ISSNHL. As such, the long-term risk for continued rapid progression of hearing loss in patients post-ISSNHL is not well-understood.

In this study, we propose that in patients with partial-to-complete recovery in hearing following ISSNHL, progression of hearing loss in affected ears would occur at a significantly faster rate than would otherwise be expected. A better understanding of hearing loss progression in ISSNHL-affected ears can allow for improved expectations management for these patients, and allow clinical otologists to think more proactively in terms of future possible interventions to improve or restore hearing. The impact of patient demographics, comorbidities, severity of initial hearing loss, and initial ISSNHL treatment strategies can furthermore aid in setting expectations for progression of future hearing loss and its management, and can lead also to improved understanding of disease etiology, potentially informing the development of novel treatments for ISSNHL.

## Materials and Methods

### Patient Selection

The Massachusetts Eye and Ear (MEE) is a tertiary referral center serving New England and adjacent regions in the Northeastern United States. The hospital has maintained comprehensive medical records for all patients during treatment at MEE, such as all audiometric evaluations performed at the facility dating back to 1994. For the purposes of this analysis, we identified patients who had received the diagnosis of ISSNHL per review of ICD9 and ICD10 billing codes from January 1, 1994 to September 30, 2018.

Assessment of hearing loss was performed by comparing the affected ear to prior audiometry if available, and otherwise by comparison with the contralateral, unaffected ear, in line with AAO-HNS guidelines (2). Audiograms were reviewed to confirm the diagnosis according to AAO-HNS criteria, where ISSNHL is defined by ≥30 dB hearing loss across three consecutive frequencies (2). In addition, a separate assessment was made by the more relaxed criterion of >10 dB difference across three consecutive frequencies to include more mild cases of ISSNHL, given that AAO-HNS guidelines do recognize a broader definition of audiometric criteria in many clinical settings. All tested frequencies from 250 Hz to 8 kHz were considered. Significance of changes in word recognition scores (WRSs) were assessed per criteria established by Halpin and Rauch 2006 (23). Audiometry to support ISSNHL diagnosis was required to be performed within 30 days preceding initial diagnosis of ISSNHL. All patients included in the study were required to have one or more additional follow-up audiometric evaluations to assess long-term hearing loss progression.

Patient charts and audiograms were reviewed to confirm ISSNHL, absence of known underlying etiology for sudden hearing loss, timing of initial diagnosis, treatment provided at time of initial diagnosis, associated symptoms at time of initial diagnosis, and baseline patient comorbidities, such as history of diabetes mellitus (DM), hypertension (HTN), hyperlipidemia (HLD), coronary artery disease (CAD), or cerebrovascular accident (CVA). All patients completed either MRI or auditory brainstem response testing to rule out retrocochlear pathology, and during initial workup did not have other associated symptoms or history to suggest any other than idiopathic etiology. Further audiometric analysis was performed to segment patients by degree of post-ISSNHL recovery as determined by best audiometry within 90 days following initial ISSNHL diagnosis, or next available audiometry if none completed within 90 days.

After defining the ISSNHL study population, an age- and sex-matched case control population was selected at 3:1 ratio from the general population at MEE; patients in the case control population were selected by screening the MEE audiometry database for patients with diagnosis code of either SNHL or mixed hearing loss, however without any other known diagnosis of any etiology for sensorineural hearing loss other than presbycusis. When more than three case control candidates were available, priority for inclusion was given to patients for whom frequency of audiometric testing most closely matched their assigned patient with ISSNHL. Hearing in case controls was matched to the study population by both four-tone pure tone average (PTA) and WRS as per the AAO-HNS Hearing Classification guidelines (24). Specifically, “good hearing” was defined as AAO-HNS Class A hearing (both PTA ≤ 30 dB and WRS ≥ 70%), with “poor hearing” the remainder (either PTA > 30 dB or WRS <70%). “Serviceable hearing” was defined as AAO-HNS Class B or better hearing (both PTA ≤ 50 dB and WRS ≥ 50%), with “non-serviceable hearing” the remainder (either PTA > 50 dB or WRS <50%). Each case control patient could be matched in terms of baseline hearing in at least one ear, with either one or both ears included for analysis depending on baseline hearing status. All case control patients with significant change in hearing over time were further reviewed in the medical record to confirm negative history of occult otologic disease that may have been missed by evaluation of diagnosis codes alone; those with history of occult otologic disease were then excluded and replaced if identified by chart review. When more than three case control candidates were available, priority for inclusion was given to the patients for whom frequency of audiometric testing most closely matched their assigned patient with ISSNHL.

A separate cohort of case controls were additionally matched by three-tone PTA (averaged across 500 Hz, 1, and 2 kHz) or word recognition criteria alone, with the remainder of screening for occult otologic disease performed in the same way as for those matched by AAO-HNS criteria. The analyses of hearing loss progression by isolated threshold and word recognition criteria could then be performed as secondary endpoints. As an isolated metric, a three-tone PTA was chosen for threshold analysis because this metric is most closely associated with speech reception thresholds and thus of highest clinical utility (25–27).

### Audiometry

Patients with ISSNHL were stratified by baseline audiometry; audiologic measurements included speech audiometry (WRS%) and threshold audiometry (dB HL). WRS was calculated using a standardized word list of monosyllables, measured as a percentage of correctly recognized words after listening to a recorded word list in quiet, typically at 70 dB or at the level at which the patient's speech intelligibility curve plateaus. Pure tone average (PTA) threshold was calculated as a four-tone average of bone conduction thresholds across 500 Hz, 1, 2, and 3 kHz frequencies as the primary threshold-based hearing metric. Good hearing was defined as PTA ≤ 30 dB and WRS ≥ 70%, equivalent to AAO-HNS Class A hearing, with hearing otherwise defined as poor. If a patient's initial audiometry was performed in the setting of sudden SNHL, however subsequent audiometry demonstrated return to good hearing within 90 days, then baseline hearing was considered to be good for purposes of baseline hearing assignment. For purpose of secondary endpoint analysis by isolated threshold and word recognition criteria, normal baseline hearing was defined as WRS ≥ 92% and a PTA threshold of ≤ 25 dB.

Progression of audiometric change was assessed both ipsilateral and contralateral to initial ISSNHL event. For patients with good hearing after recovery, the primary assigned endpoint was defined as progression to non-serviceable hearing—either increase in thresholds >50 dB or decline in WRS <50%, equivalent to AAO-HNS Class C or worse hearing. For secondary endpoint analysis, a threshold endpoint was assigned as either moderate hearing loss (PTA > 40 dB) or moderately severe hearing loss (PTA > 55 dB) in patients with baseline normal hearing, per definition by the American Speech-Language-Hearing Association. Patients with baseline mild hearing loss (PTA > 25–40 dB) were also assessed for progression to moderately severe hearing loss. In addition, for secondary endpoint analysis, WRS% endpoint was defined either as WRS <78% (significant decline from baseline normal hearing at ≥92%) or word recognition <60% (significant further decline from 78%, and also clinical threshold for consideration of cochlear implantation) (23, 28). Patients with baseline reduced WRS (78– <92%) were also assessed for progression to WRS <60%.

### Subgroup Analyses

Odds ratios (*OR*s) were calculated for patient subgroups based on age, sex, comorbidities, associated symptoms, degree of initial hearing loss, contralateral hearing status, and presence or absence of known steroid treatment at time of initial presentation. Primary endpoint for subgroup analyses was determined to be at 5 years post-ISSNHL to maintain sufficiently large sample size across all groups. Patients with less than 5 years' follow up were not included in this portion of the analysis.

### Statistics

Progression of hearing loss over time was evaluated using Kaplan–Meier (K–M) analysis, with change in hearing used to define endpoint. *OR*s for pre-defined subgroups were calculated to evaluate subgroup-specific factors and their effect. All analyses were performed in Matlab (Mathworks, Natick, MA, USA) and Excel (Microsoft Corporation, Redmond, WA, USA).

### Study Approval

Institutional Review Board (IRB) approval was obtained from the Human Studies Committee at Massachusetts Eye and Ear and Massachusetts General Hospital (IRB 17-120H), Massachusetts, United States. A written informed consent was not necessary given the observational nature of the study and no expected risk to participants from study inclusion.

## Results

### Exclusion Criteria

**Figure 1:**
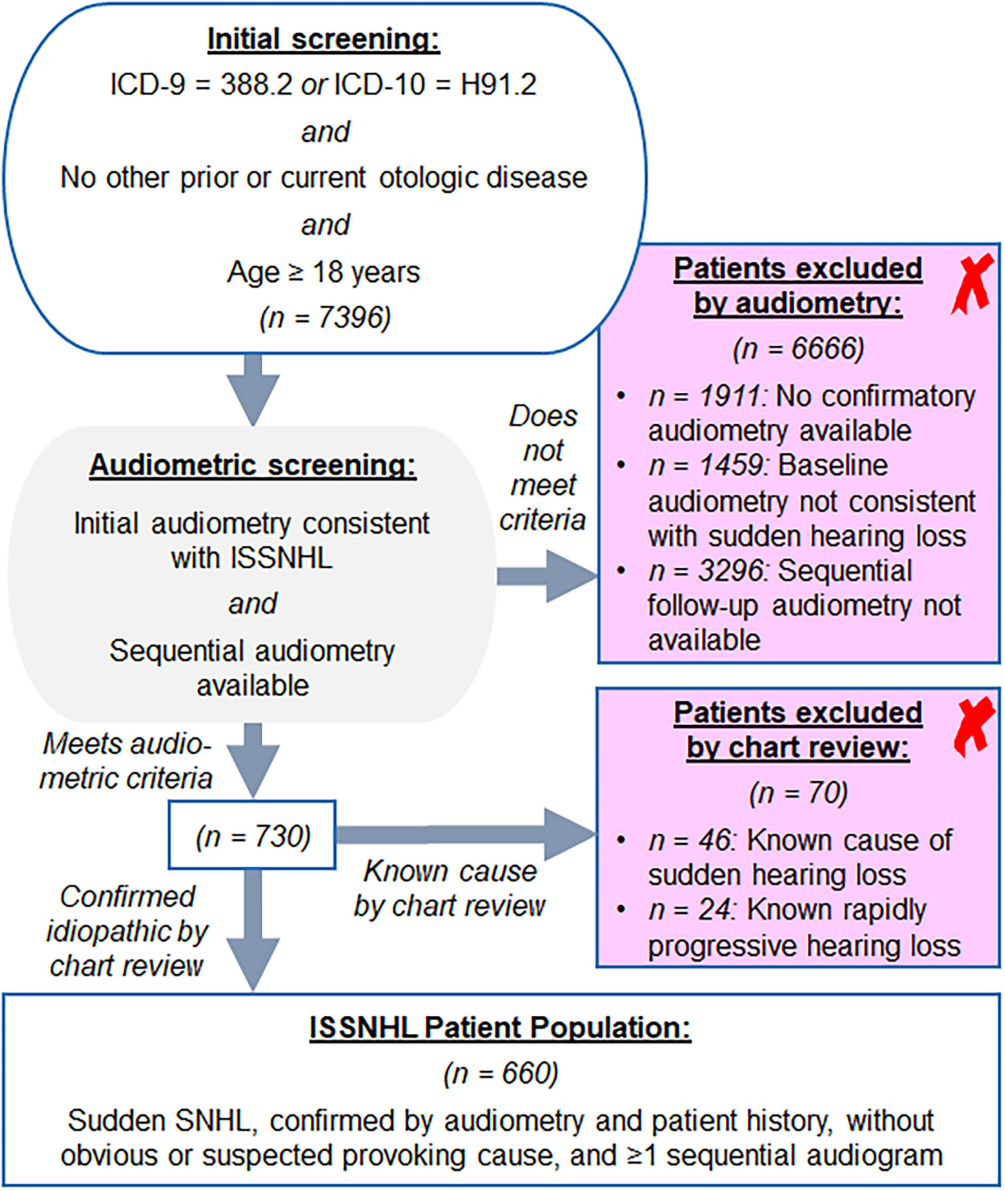
Screening approach from Mass Eye and Ear audiology database for the patients with idiopathic sudden sensorineural hearing loss (ISSNHL) to include in study, as described in section Methods.

Per review of billing codes, 7,396 patients with ISSNHL were identified. Audiometric review that included requirement for sequential audiometry after initial diagnosis yielded 730 patients age ≥ 18 years with audiometric patterns consistent with ISSNHL as described in Methods. The majority of excluded patients either had historical rather than contemporary diagnosis of sudden hearing loss, or somehow were assigned the diagnosis code despite lack of supporting audiometric data. Further chart review revealed that of these patients, 24 had rapidly progressive SNHL rather than true sudden hearing loss, while 46 had sudden SNHL due to known etiology; all 70 of these patients were excluded. Of those with known etiology for sudden SNHL, 17 were due to acoustic trauma, 13 were due to head trauma, five due to barotrauma, five due to autoimmune inner ear disease (Susac syndrome, rheumatoid arthritis, or lupus), two due to known Meniere's disease, two with recent history of ototoxic drug administration, one due to complications of chronic otitis media, and one with underlying mitochondrial disease. After exclusions noted above, a core population of 660 patients diagnosed with ISSNHL was identified as outlined in Figure 1. In patients from this population with recovery to good hearing in at least one ear, a total of 3,059 ear years were available for the analysis of long-term hearing outcomes; among the case control population, a total 8,296 ear years were available for analysis. Average interval of follow-up audiometry was 1.3 years, and the selection of age- and sex-matched case controls, as described in Methods, resulted in average interval of follow-up audiometry also of 1.3 years.

### Characteristics of the Included Patients

Clinical characteristics, demographics, associated otologic symptoms, and initial treatment of patients included in the study are given in Table 1A. The average patient age was 58.1 years, with 329 male (49.8%) and 331 female (50.2%) patients; female patients were on average 0.6 years older. HTN was reported in 130 patients (19.6%), HLD in 99 (15.0%), DM in 40 (6.1%), CAD in 24 (3.6%), and history of cerebrovascular accident (CVA) in 6 (0.9%). HTN, HLD, diabetes, and CAD were all more common in males compared with females, while prevalence of prior CVA was not different based on sex.

**Table 1A:** The demographics of a patient with idiopathic sudden sensorineural hearing loss (ISSNHL).

	**ISSNHL patient demographics**				
**Patient population**		**All patients**	**Males**	**Females**	***p*-value**
		***N* = 660**	***N* = 329**	***N* = 331**	
Age (years)	Mean	58.1	57.8	58.4	*p* = 0.58
	Range	18–95	18–88	20–95	
Pre-existing comorbidities	Hypertension	130 (19.6%)	77 (23.4%)	53 (16.0%)	*p* = 0.0003
	Hyperlipidemia	99 (15.0%)	57 (17.3%)	42 (12.6%)	*p* = 0.012
	Diabetes	40 (6.1%)	24 (7.3%)	16 (4.8%)	*p* = 0.037
	CAD	24 (3.6%)	20 (6.1%)	4 (1.2%)	*p* < 0.0001
	CVA	6 (0.9%)	2 (0.6%)	4 (1.2%)	*p* = 0.32
Treatment	Steroids PO only	350 (53.0%)	176 (53.5%)	174 (52.6%)	*p* = 0.74
	Steroids PO + IT	86 (13.0%)	48 (14.6%)	38 (11.5%)	*p* = 0.077
	Steroids IT only	48 (7.2%)	21 (6.4%)	27 (8.2%)	*p* = 0.24
	Other	46 (6.9%)	20 (6.1%)	26 (7.9%)	*p* = 0.23
	None	43 (6.5%)	21 (6.4%)	22 (6.6%)	*p* = 0.85
	Unknown	87 (13.2%)	43 (13.1%)	44 (13.3%)	*p* = 0.91
Associated symptoms	Tinnitus	424 (64.2%)	218 (66.3%)	206 (62.2%)	*p* = 0.13
	Dizziness	152 (23.0%)	68 (20.6%)	84 (25.4%)	*p* = 0.050^*^
	Vertigo	109 (16.5%)	43 (13.1%)	66 (19.9%)	*p* = 0.0018

*CAD, Coronary artery disease; CVA, Cerebrovascular accident; IT, intratympanic*.

* *p = 0.0497 when taken to an additional significant figure, thus < 0.05*.

In conjunction with onset of sudden hearing loss, the majority of patients reported new onset or worsening of tinnitus (64.2%), while new onset or worsening of either dizziness (23.0%) or vertigo (16.5%) were less common; female patients, however, were more likely to report either dizziness or vertigo compared with males. Among the male population 218 (66.3%) reported episodes of tinnitus vs. 206 (62.2%) in the female group, but this finding was not significant. Treatment for the majority of the patients consisted of isolated oral steroids (53.0%), while both PO and IT steroids (13.0%) and only IT steroids (7.2%) were prescribed less often. While a wide range of protocols for treatment was used, the most common form of oral steroids involved a prednisone burst and taper (60 mg daily for 10–14 days, followed by 5-day taper), or a series of 3–4 intratympanic injections of dexamethasone 10 mg/ml, typically with 0.5–0.8 ml volume per successful injection. Combination oral and IT steroid therapy usually involved initial prednisone treatment followed by salvage IT injections if less than complete recovery was achieved with prednisone alone. An additional 6.9% of the patients received other treatment, such as antiviral drugs, hyperbaric oxygen therapy (HBOT), or antibiotics. No treatment was provided in 6.5% of patients, and in 13.2% of patients, treatment was unable to be determined based on the medical record.

Table 1B describes severity and laterality of initial ISSNHL. Males were more likely to present with bilateral sudden hearing loss, with 10.6% of males vs. 6.9% of females affected in this manner, however not meeting significance for this difference. The severity of hearing loss followed a bimodal distribution with the most common levels of severity being either a significant shift but still within the “normal” hearing range, or else complete loss of useful hearing. This pattern was maintained regardless of measurement by threshold shift or change in word recognition.

**Table 1B:** Distribution of ISSNHL initial severity.

	**Distribution of ISSNHL initial severity**				
**Patient population**		**All patients**	**Males**	**Females**	***p*-value**
		***N* = 660**	***N* = 329**	***N* = 331**	
Hearing loss laterality	Right	305 (46.2%)	144 (43.8%)	161 (48.7%)	*p* = 0.42
	Left	297 (45.0%)	150 (45.6%)	147 (44.4%)	
	Bilateral	58 (8.8%)	35 (10.6%)	23 (6.9%)	
Severity of initial hearing loss	Thresholds
	Normal	259 (39.3%)	126 (38.3%)	133 (40.1%)	*p* = 0.87
	Mild	86 (13.0%)	44 (13.4%)	42 (12.7%)	
	Moderate	59 (8.9%)	26 (7.9%)	33 (10.0%)	
	Mod. severe	70 (10.6%)	31 (9.4%)	39 (11.8%)	
	Severe	61 (9.2%)	34 (10.3%)	27 (8.2%)	
	Profound*	125 (19.0%)	68 (20.7%)	57 (17.2%)	
	Word recognition				
	≥92% (Normal)	229 (34.7%)	109 (33.1%)	118 (35.9%)	*p* = 0.75
	78– <92%	113 (17.1%)	62 (18.9%)	51 (15.5%)	
	60– <78%	50 (7.6%)	28 (8.5%)	22 (6.7%)	
	<60%*	268 (40.6%)	130 (39.5%)	138 (41.9%)	

Threshold classification per American Speech-Language-Hearing Association; the categories for significant differences in word recognition per Halpin and Rauch 2006 (23); For the patients meeting criteria for ISSNHL bilaterally, hearing loss is shown for the worse ear only; ^*^includes unmeasurable thresholds or word recognition unable to test secondary to profound hearing loss.

Table 2 describes characteristics of patients post-recovery from unilateral ISSNHL, segmented by degree of post-recovery hearing in ipsilateral vs. contralateral ears. Among the 471 patients with good hearing in ISSNHL-contralateral ear, 187 (39.7%) recovered to good hearing in the ISSNHL-ipsilateral ear as well, while 284 had incomplete hearing recovery. Among the 131 patients with baseline poor hearing in the ISSNHL-contralateral ear, 25 (19.1%) recovered to good hearing in the ipsilateral ear, while 106 had incomplete hearing recovery ipsilaterally. The degree of post-ISSNHL hearing recovery in the ipsilateral ear was significantly (*p* < 0.0001) associated with hearing status in the contralateral ear.

**Table 2:**
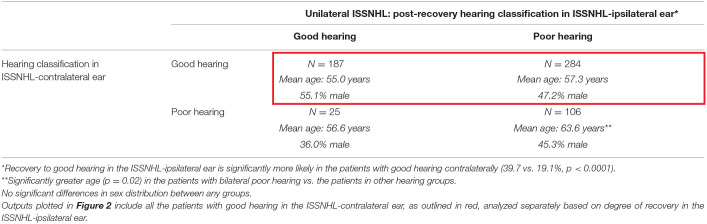
The demographics in the patients with audiometrically-confirmed unilateral ISSNHL, segmented by degree of hearing recovery post-ISSNHL.

Table 3 describes characteristics of patients post-recovery from bilateral ISSNHL, segmented by degree of post-recovery hearing. Among these 58 patients, 7 (12.1%) recovered to good hearing bilaterally, while 18 (31.0%) recovered to good hearing in one ear only, and 33 (56.9%) did not recover to good hearing in either ear. Overall rates of recovery to good hearing in any ear were lower than in patients with unilateral ISSNHL.

**Table 3:**
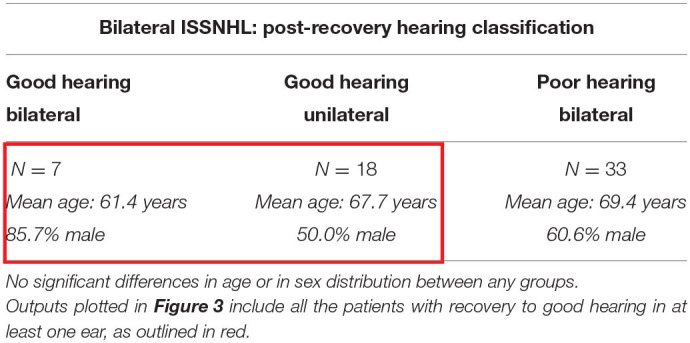
The demographics in the patients with audiometrically-confirmed bilateral ISSNHL, segmented by degree of hearing recovery post-ISSNHL.

### Time to Hearing Loss Progression in Patients With Recovery to Good Hearing Post-ISSNHL

**Figure 2:**
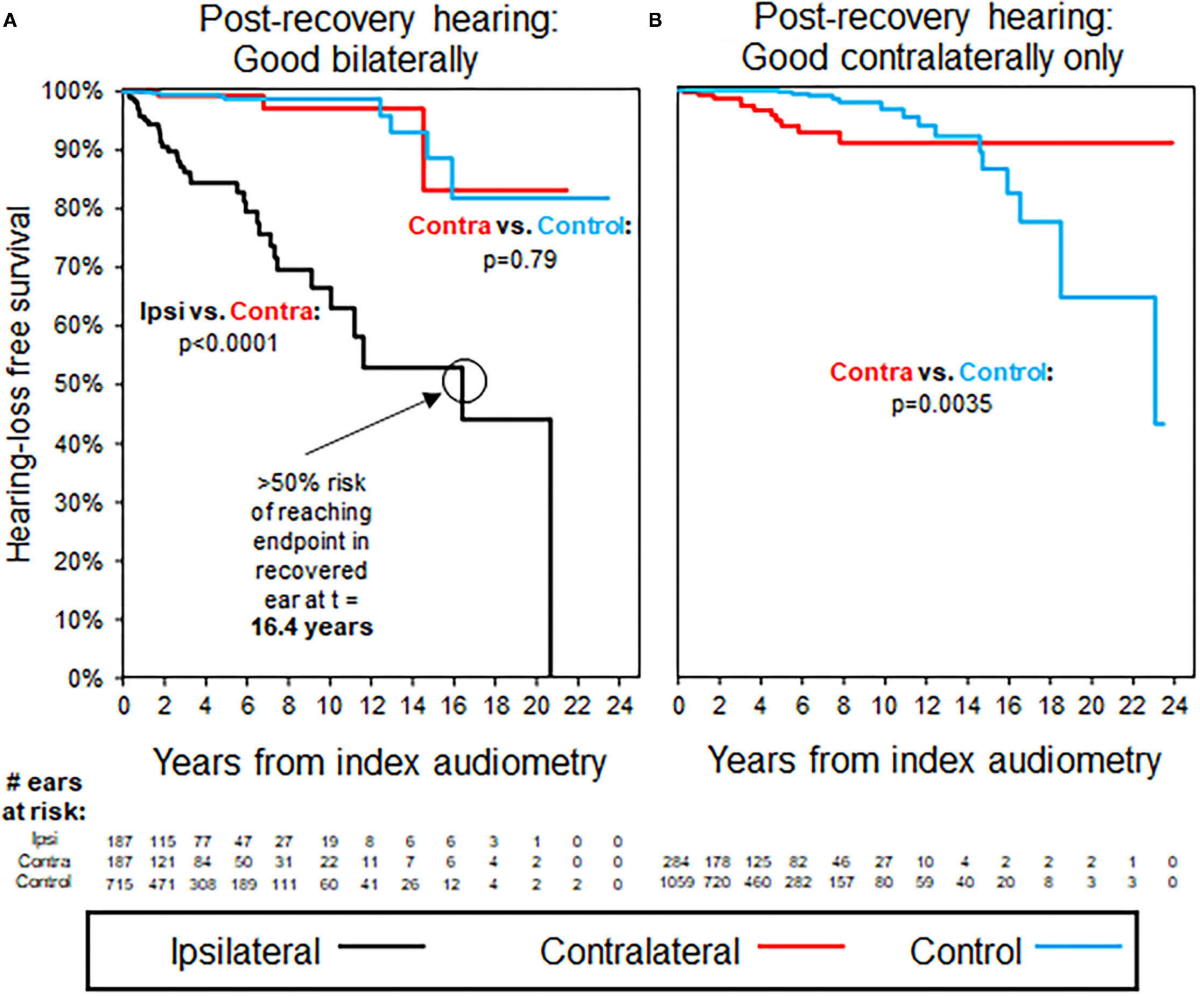
Kaplan–Meier curves for hearing loss progression in the patients with unilateral ISSNHL and good baseline hearing in the ISSNHL-contralateral ear; as shown in Table 2 for demographics. **(A)** The patients with good hearing bilaterally, endpoint non-serviceable hearing. **(B)** The patients with good hearing in ISSNHL-contralateral ear only, endpoint non-serviceable hearing. All the control ears with good hearing.

Results of K–M survival analysis in Figure 2A shows that patients with recovery to good hearing after ISSNHL demonstrated significantly faster decline in hearing compared with both contralateral ears and compared with age- and sex-matched case controls (*p* < 0.0001). In these recovered ears, median time to reach endpoint of non-serviceable hearing (either PTA > 50 dB or WRS <50%) was 16.4 years. Average follow-up interval between audiograms was 1.3 years for these patients, and also for selected case controls.

### Time to Hearing Loss Progression in Contralateral Ears Without Ipsilateral Recovery to Good Hearing

Figure 2B shows that patients without ipsilateral recovery to good hearing after ISSNHL, but with good hearing still in the contralateral ear, were significantly more likely than controls to demonstrate a decline in hearing in the remaining better-hearing ear. After separating early, K–M hazard ratio curves do cross again at 14.6 years follow-up, however, at this time point only 37 ears out of an initial 1,343 (2.8%) are still at risk, and the significantly higher time-adjusted risk of reaching endpoint in ISSNHL-contralateral ears vs. controls (*p* = 0.0035) is driven primarily by events earlier in follow-up. Average follow-up interval between audiograms was 1.3 years for these patients, and also for selected case controls.

### Time to Hearing Loss Progression in Patients With Bilateral ISSNHL

**Figure 3:**
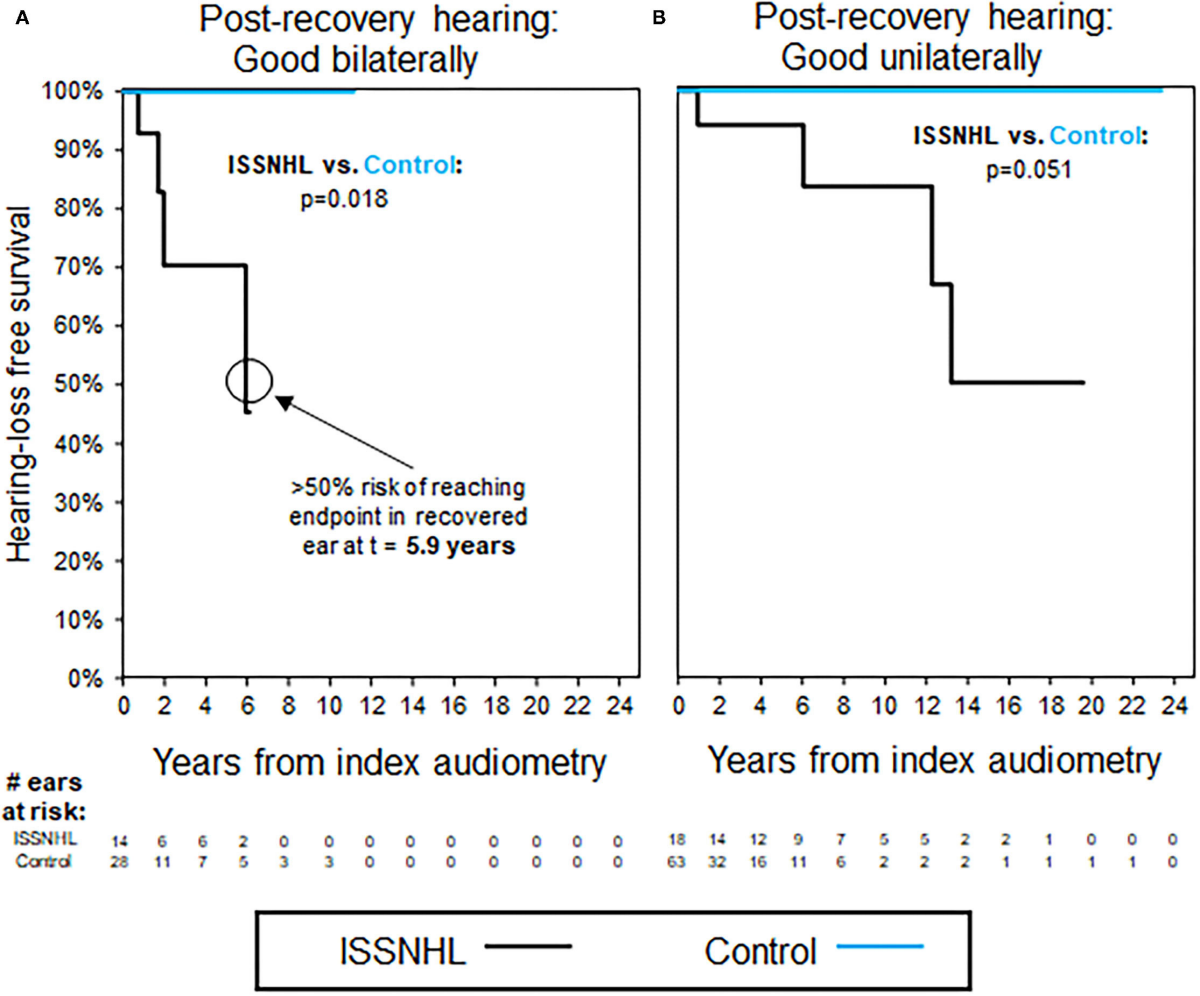
Kaplan–Meier curves for hearing loss progression in the patients with bilateral ISSNHL and recovery to good hearing in at least one ear; as shown in Table 3 for demographics. **(A)** The patients with good hearing bilaterally, endpoint non-serviceable hearing. **(B)** The patients with good hearing in one ear only, endpoint non-serviceable hearing. All the control ears with good hearing.

Figure 3 shows trends in hearing loss progression for patients with bilateral ISSNHL and recovery to good hearing in either both ears (Figure 3A) or in only one ear (Figure 3B). In patients with bilateral ISSNHL and bilateral recovery, hearing loss progression is even faster than in the unilateral hearing loss group, with median time to reach non-serviceable hearing of only 5.9 years (Figure 3A). In patients with recovery to good hearing in only one ear, hearing loss progression occurs at a similar rate to that seen in patients with unilateral ISSNHL (Figure 3B). Average follow-up interval between audiograms was 1.3 years for these patients, and also for selected case controls.

### Risk Factor Assessment for Hearing Loss Progression After Recovery to Good Hearing Post-ISSNHL

**Figure 4:**
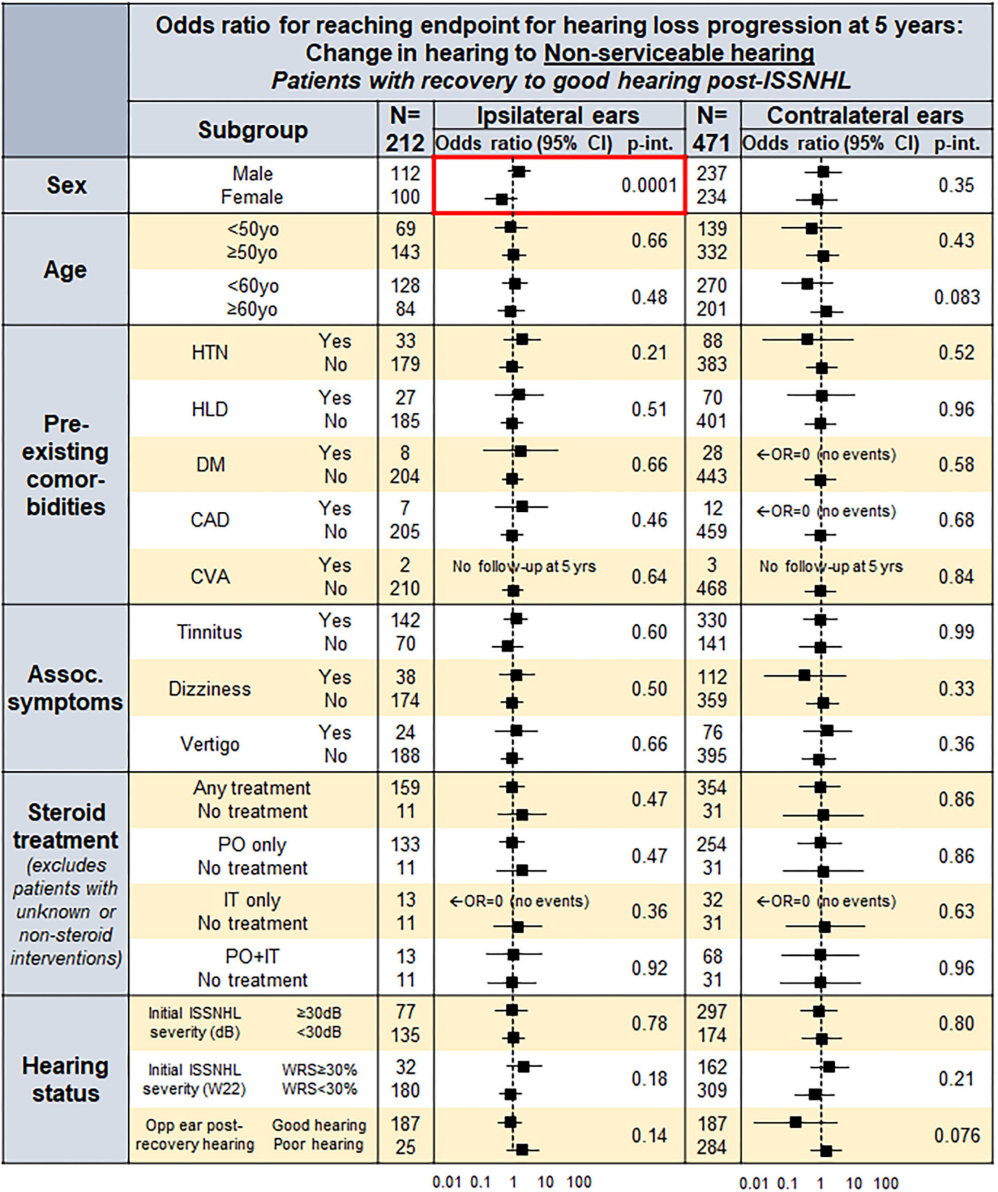
A forest plot for odds ratio (*OR*) to reach endpoint by endpoint non-serviceable hearing in both the ISSNHL-ipsilateral and contralateral ears at 5 years, in the patients with post-ISSNHL recovery to good hearing in the respective ear.

Figure 4 outlines the influence of patient factors for reaching hearing loss endpoint at 5 years post-ISSNHL, both for ISSNHL-ipsilateral (left column) and ISSNHL-contralateral (right column) ears with good hearing at baseline. Male sex was found to be a consistently strong predictor for greater odds of progression to non-serviceable hearing in both ISSNHL-ipsilateral and contralateral ears, while no other patient demographics features, comorbidities, ISSNHL-associated symptoms, ISSNHL drug therapy, or hearing status in the opposite ear were found to significantly affect risk of hearing loss progression.

### Additional Analyses in Appendix

Further analyses seek to classify patients by using baseline hearing in terms of isolated threshold or word recognition criteria, rather than combined classifications. General trends are similar and demonstrate robustness of findings across a range of hearing loss endpoints, and can be found in the Appendix.

## Discussion

### Key Findings

To our knowledge, this study represents the largest-to-date assessment of long-term hearing loss progression in patients with audiometrically-confirmed ISSNHL, and shows that continued progression of hearing loss following ISSNHL is faster than otherwise expected. In patients with incomplete ipsilateral recovery in hearing post-ISSNHL, an increased risk to long-term hearing was seen in contralateral ears as well. Subgroup analyses demonstrated that male sex was associated with significantly greater odds of hearing loss progression at 5 years post-ISSNHL recovery, both in ISSNHL-ipsilateral and contralateral ears. Other patient factors, such as age, comorbidities, drug therapy, and opposite ear hearing, were not significantly associated with odds of hearing loss progression.

### Setting Expectations for Hearing Loss Progression Post-ISSNHL Recovery

A minority of patients in our study demonstrated recovery to good hearing post-ISSNHL. Progression of hearing loss was later observed in these recovered ears, with median risk of progression to non-serviceable hearing by 16.4 years. Even contralateral ears, unaffected by initial sudden hearing loss, were shown to be at increased risk of hearing loss progression in patients with incomplete recovery in ISSNHL-ipsilateral hearing. The greater risk of hearing loss progression ipsilaterally may be due to continued presence of systemic risk factors or repeated exposure to the cause of the initial insult, or additionally due to the recovered ear maintaining a certain level of residual “hidden” hearing loss that is not detected by conventional audiometry; in this case, the loss and recovery of hearing with ISSNHL would be considered as similar to the temporary threshold shift seen in acoustic trauma (29). Increased risk of hearing loss in contralateral ears, on the other hand, would be more likely due only to persistence of systemic risk factors or repeated exposure to causative agents for ISSNHL, and so should demonstrate comparatively less long-term risk to contralateral hearing—although as can be seen in Figure 2B, the risk is not zero. Likelihood for long-term hearing loss progression did not differ significantly based on whether initial hearing loss was unilateral or bilateral.

Recent studies have demonstrated the role of secreted factors in causing hearing loss in pathologies such as vestibular schwannoma, which can present with sudden SNHL and for which the audiometric hallmark is disproportionate loss in word recognition relative to threshold shift; undiagnosed Meniere's and autoimmune ear disease may also increase risk to bilateral hearing (27, 30, 31). While none of the patients in the current study were known to have other otologic disease at the time of initial evaluation for ISSHNL, sudden hearing loss can often serve as the initial presentation for an underlying inflammatory or autoimmune mechanism. Given the increased risk to ISSNHL-contralateral ears in patients with incomplete recovery in ISSNHL-ipsilateral hearing, it is possible in these patients that secreted inflammatory markers could explain the faster rate of hearing loss progression bilaterally, similar to other known inflammatory pathologies associated with sudden SNHL (27, 32–34).

Post-ISSNHL, all patients should thus be counseled of the persistent risk to hearing, both ipsi- and contralaterally. While the greatest risk seen is the accelerated ipsilateral hearing loss progression for patients with recovery to good hearing, contralateral ears continue to be at risk as well, particularly in those patients with incomplete recovery in ipsilateral hearing.

### Implications of Key Findings From the Subgroup Analysis

These findings do not demonstrate significance of patient comorbidities or risk factors that have previously been shown to increase risk for sudden hearing loss, such as vasculopathic and cardiovascular risk factors (10, 11). Figure 4 demonstrates that in ISSNHL-ipsilateral ears with recovery to good hearing, risk of hearing loss progression is independently higher, although non-significant, for all key cardiovascular risk factors evaluated—HTN, diabetes, HLD, and CAD. Only male sex, however, was found to significantly increase risk of hearing loss progression independent of other comorbidities; it must be noted that males in our study were much more likely to have been previously diagnosed with these risk factors, and that the significance of male sex as a predictor for hearing loss progression disappeared once controlling for these comorbidities. This suggests that risk stratification should not treat male sex as an independent risk factor, but rather focus on the underlying comorbidities that are simply more common in males, even if no one single predisposing comorbidity is by itself significant.

Of note, males are actually over-represented in the patient group with recovery to normal hearing bilaterally—this finding could be explained by a greater degree of reversibility in mechanisms that are predominantly common in the male population, such as the cardiovascular risk factors noted above, while other mechanisms that are relatively more prevalent in the female population are less likely to be reversible (10). While similar trends are seen in the bilateral ISSNHL group, with males representing 6/7 patients with bilateral hearing loss and full recovery, this trend is not significant.

No correlation was found among steroid treatment, route of steroid administration, or degree of initial hearing loss with risk of long-term hearing loss progression, and regardless of hearing loss endpoint evaluated—this may be because treatment choice is often confounded by other factors, such as patient steroid tolerance, and because the broad acceptance of steroids for sudden hearing loss led to very few patients not receiving some form of steroid therapy. Lack of effect seen with regard to initial degree of threshold shift is particularly interesting to note, given that a key component to the AAO-HNS guidelines defining ISSNHL is loss of ≥30 dB across three sequential frequencies, with previous research demonstrating a “floor effect” for improvement in hearing with small thresholds losses (2, 35). On the other hand, initial loss in ipsilateral word recognition ≥30%, although never reaching significance as an isolated patient factor, did demonstrate a consistent trend toward increased risk of hearing loss progression both in ipsilateral and contralateral ears. Taken together, these findings may support a broader definition of ISSNHL diagnosis that would be less strict in terms of threshold shift and more prominently feature change in word recognition as the key diagnostic criterion.

Regarding relative risk due to symptoms associated with initial hearing loss, no significant associations were found between symptoms and risk of hearing loss progression. This is somewhat surprising given that tinnitus at least has previously been shown to be associated with worse prognosis in sudden hearing loss (10, 11).

### Relative Value of Hearing Loss Metrics

Alternate classification of hearing by isolated threshold or word recognition metrics showed that directionality and significance of results were consistent regardless of which metrics were used and how endpoints were defined. The details of these analyses can be found in the Appendix.

### Limitations

Due to the retrospective study design, availability of records was incomplete for some patients, and the interval and duration of follow-up audiometry inconsistent as well—K–M survival analysis was able to account for the effect of variable follow-up by matching average follow-up audiometry intervals between study patients and case controls, however cannot account for differences in total duration of follow-up among the individual patients. In many patients first seen at our institution for sudden hearing loss, no prior audiometry was available to assess baseline hearing, thus necessitating comparison with the contralateral ear to assess degree of initial hearing loss—as a result, it is possible that some cases of bilateral sudden hearing loss were excluded from the analysis. Mass Eye and Ear is a tertiary referral center—many patients whose initial diagnosis of ISSNHL was based on outside audiometry may not have had index audiometric data included in our audiology database, and thus would not have passed audiometric screening criteria to confirm the diagnosis and be included for analysis. Many patients who were referred to Mass Eye and Ear for evaluation likely returned to outside care after consultation for ISSNHL, and this undoubtedly contributed to the high initial screen out rate for patients without sequential audiometry after initial diagnosis. Additionally, certain patient factors, such as lack of steroid treatment, or history of CAD or CVA, were not present at sufficient frequency within the population to assess impact on long-term hearing outcomes with an appropriate level of confidence.

Our analysis focuses on hearing loss progression only in ears with good hearing post-ISSNHL, either ipsi- or contralaterally. Progression of hearing loss in ears with incomplete recovery in hearing (either PTA > 30 dB or WRS <70%) was not assessed due to lower clinical relevance, since the vast majority of these patients already did not have useful hearing in the relevant ear.

### Case Control Group

Of particular interest to this analysis is the selection of appropriate case controls—patients with SNHL but no other known sensorineural otologic history and thus presumed presbycusis, as well as a similar interval for follow-up audiometry compared with the study population. A potential selection bias could be present within the case control group if patients with occult otologic history were included, and if this history were not evident based on superficial review of diagnostic codes alone. To minimize this bias, we performed detailed chart review on any case control patients who demonstrated significant change in hearing over time, and replaced those with other otologic histories with “clean” controls for whom non-ISSNHL otologic disease burden was equivalent to the study population, as detailed in the Methods section. This finding reinforces the continued importance of detailed chart review in the context of large database analyses, since over-reliance on the “face value” of historical diagnosis codes can easily overlook key components to the patient history.

## Conclusions

These findings represent to date the largest study tracking long-term bilateral hearing outcomes in patients with ISSNHL. Our findings definitively demonstrate an increased risk of hearing loss progression in these patients, both in ISSNHL-ipsilateral and contralateral ears. Male sex was the only significant patient factor associated with the increased risk of hearing loss progression, however, this effect was confounded by greater incidence of HTN, HLD, diabetes, and cardiovascular disease in the male patient population. All ISSNHL patients should be counseled on the increased risk of accelerated long-term hearing loss progression, with primary concern for hearing in the ISSNHL-ipsilateral ear in patients with full recovery and additionally for the contralateral ear in patients with incomplete recovery. Additional care should be taken to avoid other otologic insults, such as acoustic trauma or potentially ototoxic medications in these patients, and guidance should be provided for optimal management of modifiable risk factors such as HTN and diabetes.

Previous research indicates that secreted factors may play a role in accelerated hearing loss progression in patients with vestibular schwannoma (VS), with potential for long-distance percolation through CSF or blood given effects seen in VS-contralateral ears (27). Immune or inflammatory-mediated processes uniquely affecting the inner ear may also play a role. Understanding key characteristics of these potentially relevant mechanisms, and the interplay between them, can help guide future research to better characterize and evaluate the composition and role played by each, and their relative contribution to etiologies of progressive hearing loss, such as we have identified here for patients post-ISSNHL.

Improved understanding of causes and treatments for ISSNHL will require better understanding of disease pathophysiology, which is complicated by the likely multifactorial nature of the disease (2). We build on the results of prior studies to show that no single vasculopathic or cardiovascular disease risk factor is associated convincingly with hearing loss progression in these patients, but that a patient population with higher levels of these risk factors can be at higher risk, such as males in our study. Prospective evaluation of new patient cases of ISSNHL can explore in greater detail the role of a greater range of factors in ISSNHL long-term outcomes.

## Data Availability Statement

The primary dataset includes service dates for certain procedures which are considered protected under HIPAA guidelines, and as such cannot be shared publicly. Datasets can be made available upon request to interested parties. Requests to access these datasets should be directed to kstankovic@stanford.edu.

## Ethics Statement

This study involving human participants was reviewed and approved by Human Studies Committee at Massachusetts Eye and Ear and Massachusetts General Hospital (IRB 17-120H). Written informed consent for participation was not required for this study in accordance with the national legislation and the institutional requirements.

## Author Contributions

KS conceived the project and supervised all the work. SE and KS designed the study. SE and JV collected data from patient charts. SE wrote code for data analysis. Primary analysis and interpretation were then performed by SE and JV, with input from all authors. SE, JV, and KS wrote the manuscript. All authors edited the manuscript and approved the final version.

## Funding

This work was supported by the National Institute on Deafness and Other Communication Disorders (R01DC015824), the Nancy Sayles Day Foundation, the Zwanziger Foundation, the Barnes Foundation, and the Sheldon and Dorothea Buckler (KS).

## Conflict of Interest

The authors declare that the research was conducted in the absence of any commercial or financial relationships that could be construed as a potential conflict of interest.

## Publisher's Note

All claims expressed in this article are solely those of the authors and do not necessarily represent those of their affiliated organizations, or those of the publisher, the editors and the reviewers. Any product that may be evaluated in this article, or claim that may be made by its manufacturer, is not guaranteed or endorsed by the publisher.
