# Supplementary figures and images for: Accelerated Long-Term Hearing Loss Progression After Recovery From Idiopathic Sudden Sensorineural Hearing Loss
Source: Front Neurol. 2021 Dec 8;12:738942. doi: 10.3389/fneur.2021.738942 (PMC8693444; doi:10.3389/fneur.2021.738942)

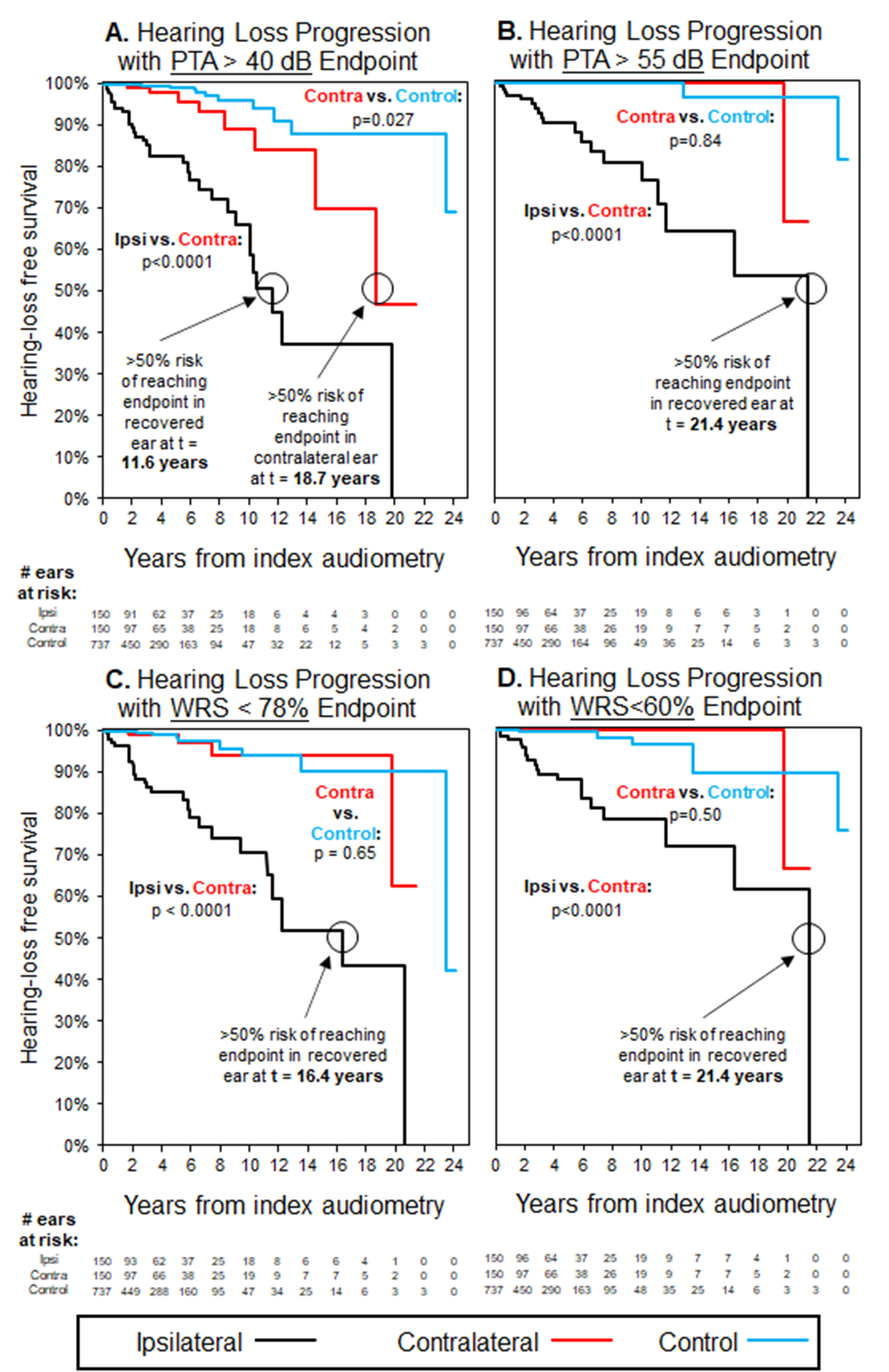

Supplement: Supplementary file 1 [file Image_1.TIF]

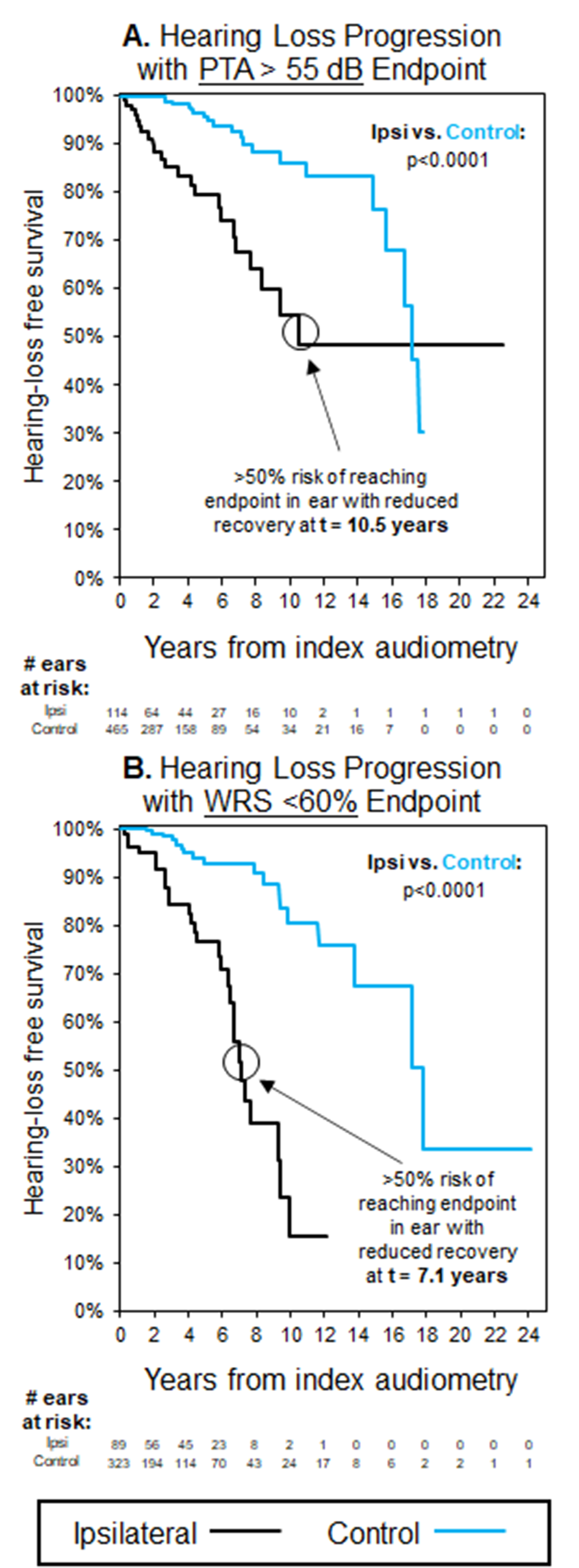

Supplement: Supplementary file 2 [file Image_2.TIF]

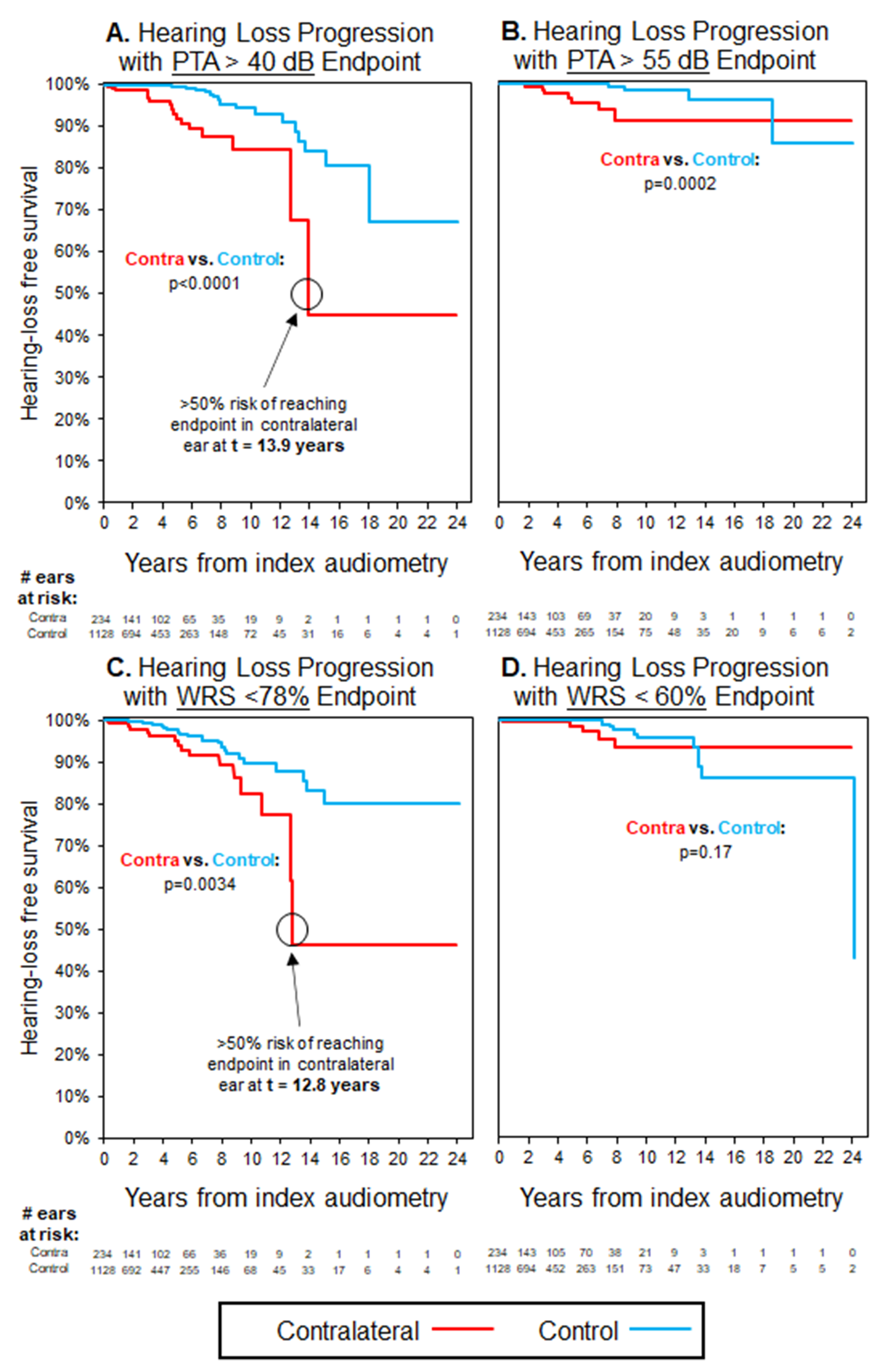

Supplement: Supplementary file 3 [file Image_3.TIF]

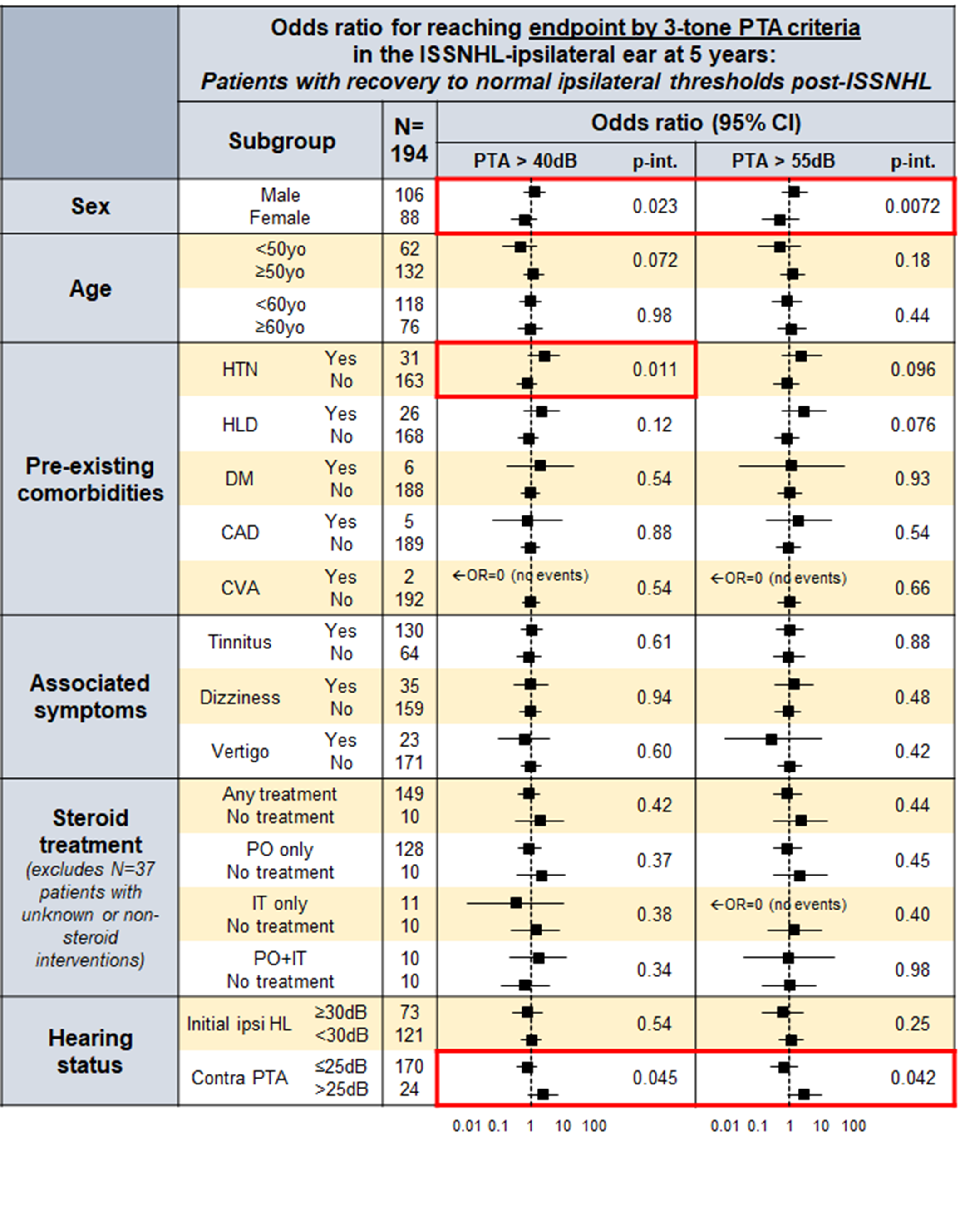

Supplement: Supplementary file 4 [file Image_4.TIF]

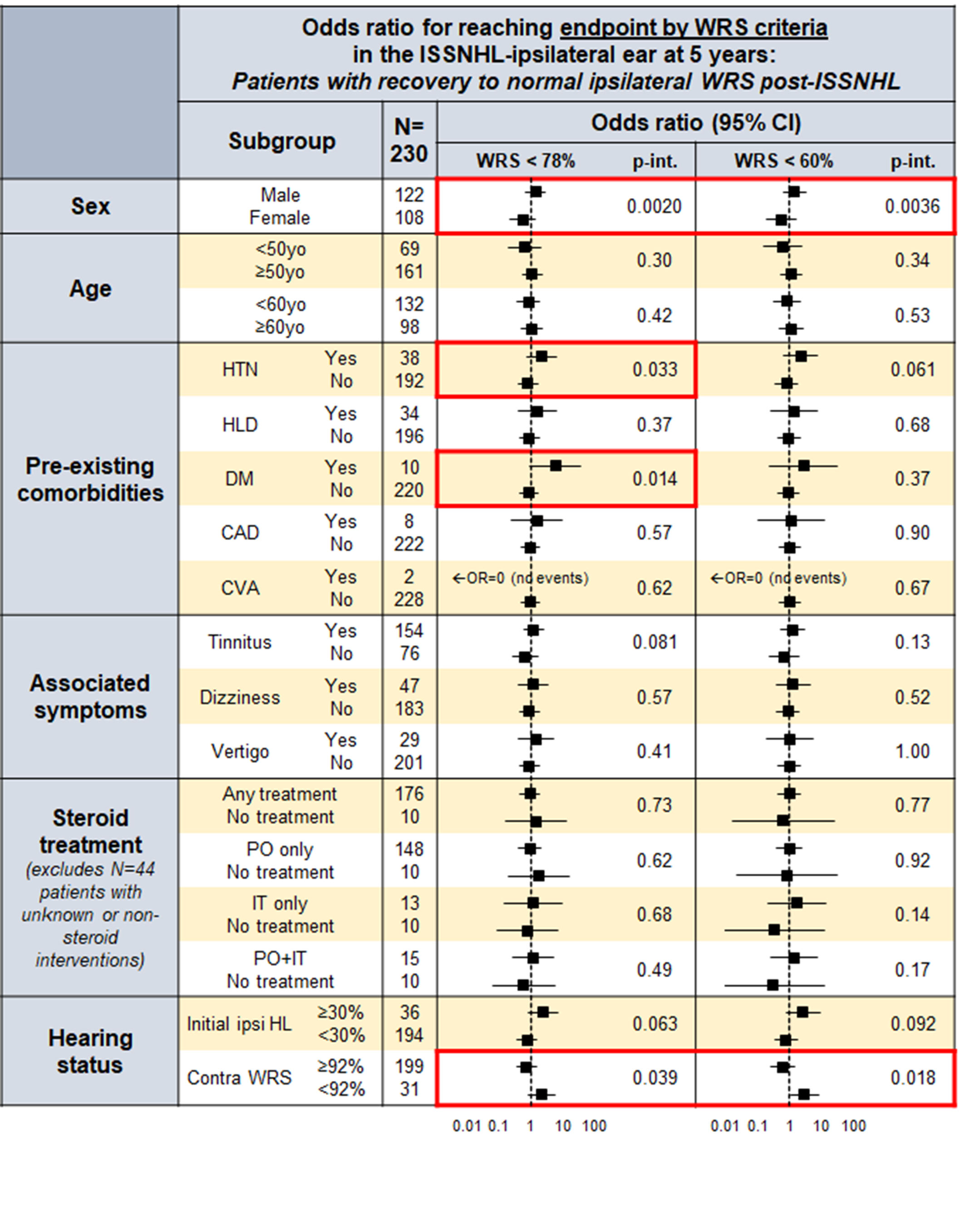

Supplement: Supplementary file 5 [file Image_5.TIF]

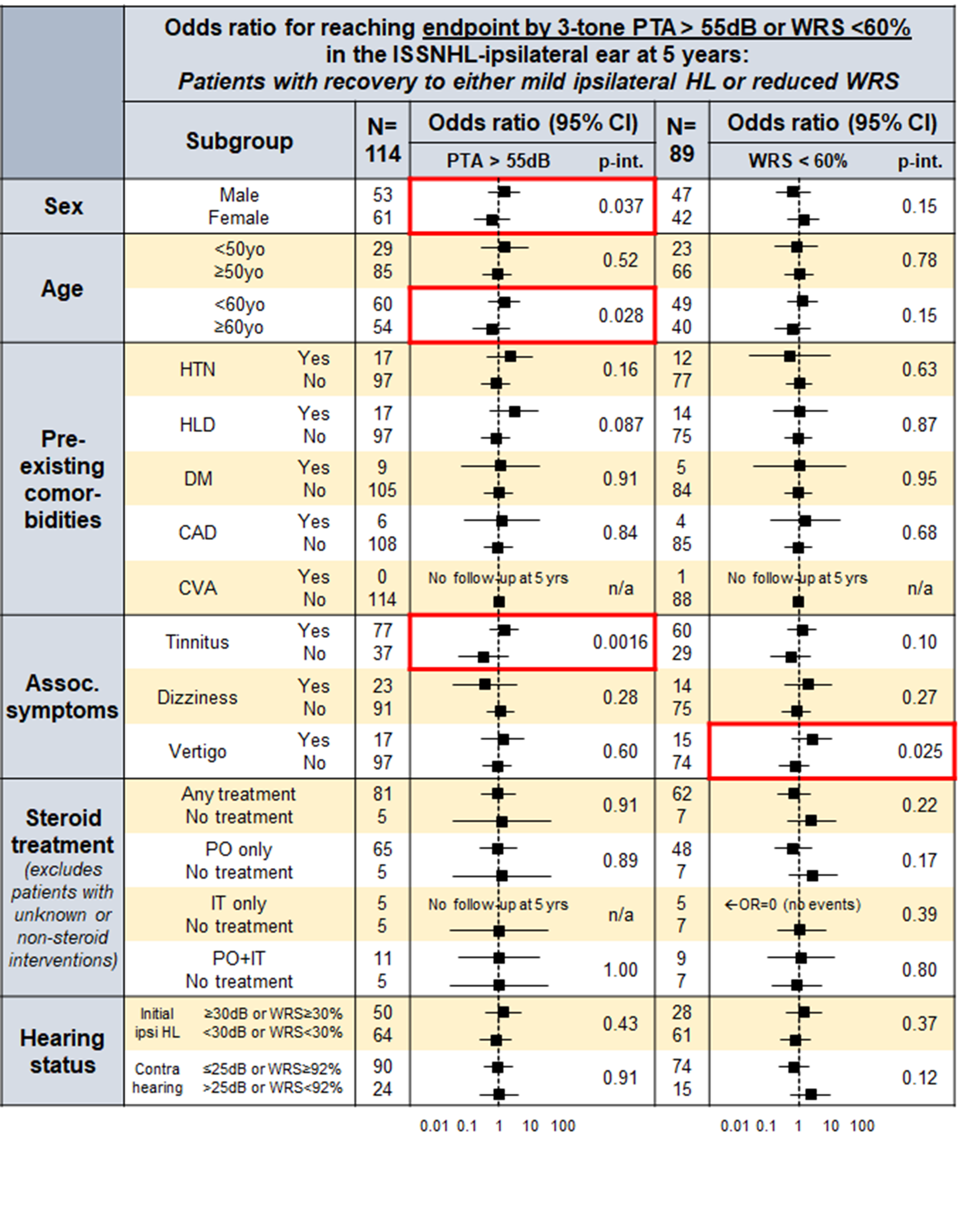

Supplement: Supplementary file 6 [file Image_6.TIF]

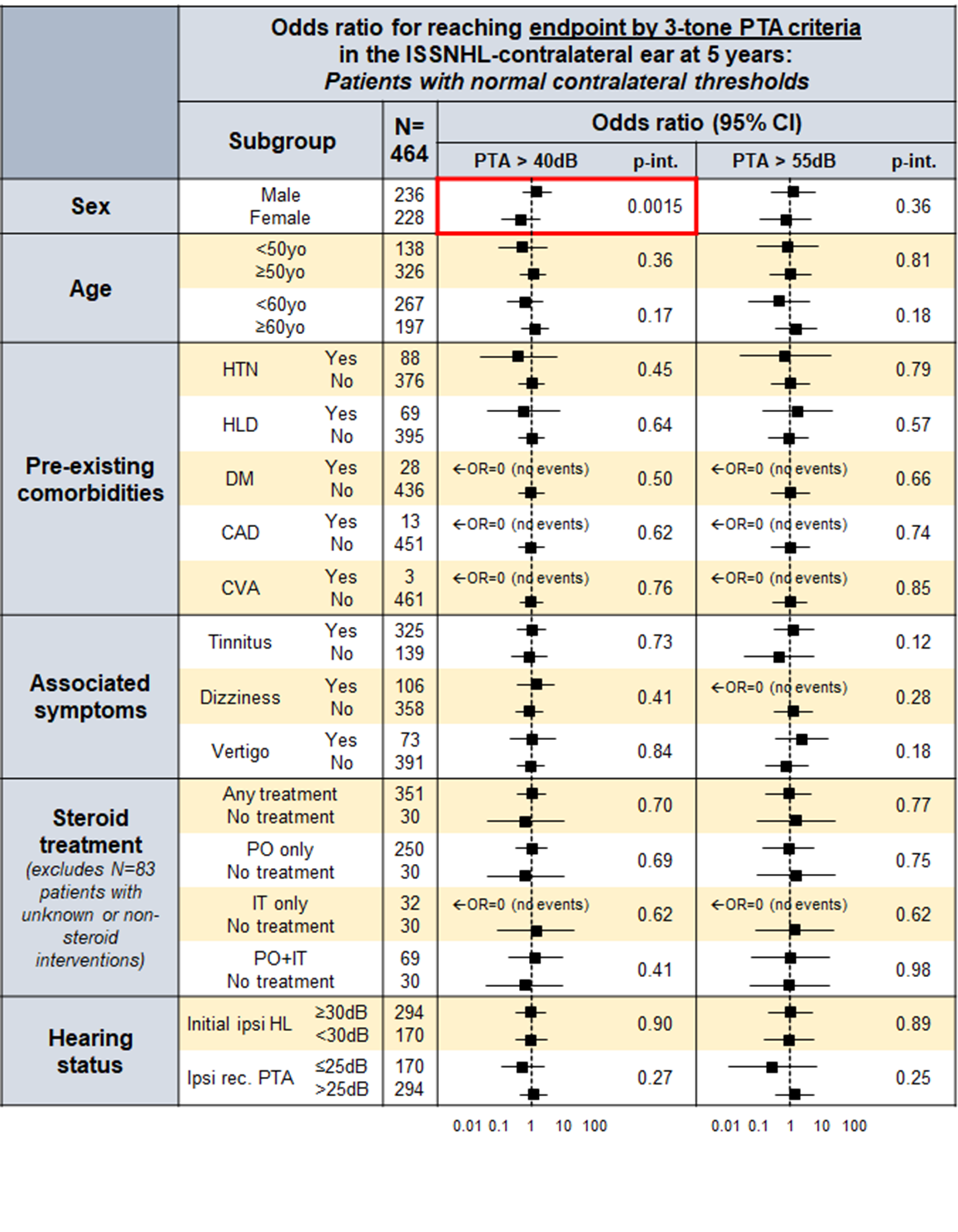

Supplement: Supplementary file 7 [file Image_7.TIF]

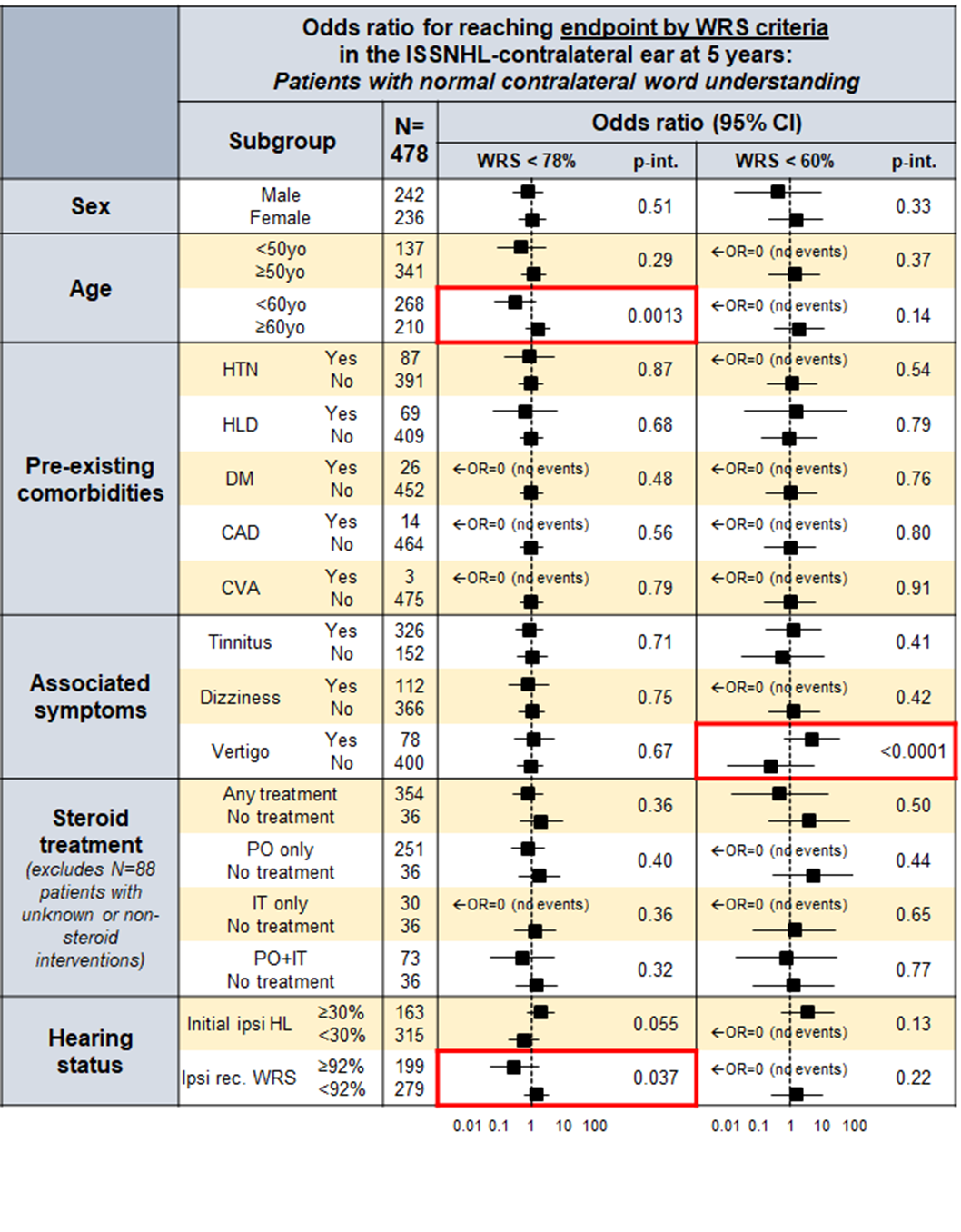

Supplement: Supplementary file 8 [file Image_8.TIF]
