# Supplementary material for: Accelerated Long-Term Hearing Loss Progression After Recovery From Idiopathic Sudden Sensorineural Hearing Loss
Source: Front Neurol. 2021 Dec 8;12:738942. doi: 10.3389/fneur.2021.738942 (PMC8693444; doi:10.3389/fneur.2021.738942)
Supplement: Supplementary file 9 [file Data_Sheet_1.docx]

Supplementary Material

**Methods**

A separate cohort of case controls were additionally matched by three-tone PTA (averaged across 500Hz, 1kHz and 2kHz) or word understanding criteria alone. Analyses of hearing loss progression by these criteria were performed as secondary endpoints. As an isolated metric, three-tone PTA was chosen because this metric is most closely associated with speech reception thresholds and thus of highest clinical utility (1-3).

For secondary endpoint analysis, threshold endpoint was assigned as either moderate hearing loss (PTA >40dB) or moderately severe hearing loss (PTA >55dB) in patients with baseline normal hearing, per definition by the American Speech-Language-Hearing Association. Patients with baseline mild hearing loss (PTA >25-40dB) were also assessed for progression to moderately severe hearing loss. Also for secondary endpoint analysis, WRS% endpoint was defined either as WRS < 78% (significant decline from baseline normal hearing at ≥92%) or word understanding <60% (significant further decline from 78%, and also clinical threshold for consideration of cochlear implantation) (4, 5). Patients with baseline reduced WRS (78-<92%) were also assessed for progression to WRS<60%.

**Results**

*Demographics in unilateral ISSNHL patient segmented by isolated threshold or word understanding criteria*

Table S1 describes characteristics of patients post-recovery from ISSNHL, among patients with normal hearing in the contralateral (unaffected) ear – these are patients that would be expected, with a full recovery, to be able to return to normal baseline hearing in the ISSNHL-ipsilateral ear as well. Of these patients, 170 demonstrated recovery to normal hearing in the ISSNHL-affected ear by 3-tone PTA criteria, and 199 by word understanding criteria; a total of 150 patients returned to normal baseline hearing by both PTA and word understanding. Additionally, 294 of these patients did not demonstrate recovery to normal hearing in the ISSNHL-affected ear by 3-tone PTA criteria, and 279 were unable to demonstrate recovery by word understanding; a total of 234 patients were unable to achieve recovery by either PTA or word understanding criteria, and these patients are classified as having abnormal hearing post-ISSNHL. Patients with recovery to normal ipsilateral hearing post-ISSNHL were significantly younger when evaluated by threshold hearing (54.5 vs. 57.2 years old, p=0.030); although no significant difference in age was seen when assessing by either word understanding alone or by both threshold and word understanding criteria, the trend towards younger age in recovered patients was consistent. Patients with recovery to normal ISSNHL-ipsilateral hearing were also significantly more likely to be male, regardless of metric used to assess hearing loss (p=0.010 by threshold criteria, p=0.011 by WRS criteria, p=0.028 by combined criteria).

In order to more precisely evaluate patients with partial hearing recovery post-ISSNHL, Table S2 describes as well the patient populations in which post-ISSNHL hearing recovery reaches the range of mild hearing loss (PTA>25-40dB) or reduced word understanding (78-<92%). In this table, contralateral hearing status is not used to restrict the patient population, given the difficulty in matching ipsilateral and contralateral hearing status in a patient with only a partially-recovered ear; patient hearing only in the ISSNHL-ipsilateral ear is evaluated. Age and sex distribution of patients in this table did not differ significantly from the general population of patients with recovery to abnormal hearing in the ISSNHL-ipsilateral ear.

*Time to hearing loss progression in patients with recovery to normal hearing post-ISSNHL*

Results of Kaplan-Meier survival analysis in Figure S1 show that patients with recovery to normal hearing after ISSNHL demonstrated significantly faster decline in hearing, both in terms of thresholds and word understanding, and regardless of endpoint chosen, compared to both contralateral ears and compared to age- and sex matched case controls (p<0.0001 for reaching endpoint by moderate hearing loss, moderately severe hearing loss, WRS<78% or WRS<60%). In these recovered ears, median time to reach endpoint of moderate hearing loss (PTA>40dB) was 11.6 years, and to reach endpoint of moderately severe hearing loss (PTA>55dB) was 21.4 years, while median time to reach endpoint of WRS<78% was 16.4 years, and to reach endpoint of WRS<60% was 21.4 years. ISSNHL-contralateral ears demonstrated also a significantly higher likelihood of reaching endpoint by moderate hearing loss vs. case controls (p=0.027), with median time to reach endpoint of 18.7 years. No other differences were noted between likelihood of contralateral ears to reach endpoint compared to controls.

*Time to hearing loss progression in patients without recovery to normal hearing post-ISSNHL*

In Figure S2, Kaplan-Meier survival analysis focuses on hearing loss progression in ISSNHL-ipsilateral ears that demonstrated only partial recovery in hearing, either to mild hearing loss (Figure S2A) or to WRS 78-<92% (Figure S2B). These ears are compared to case controls with baseline hearing matched either by thresholds or word understanding; contralateral ears are not included, since with rare exceptions these patients did not have matched hearing contralaterally. Survival analysis demonstrates significantly less time to reach endpoint by either moderately severe hearing loss or WRS<60% (p<0.0001 for both). Median risk of reaching endpoint in these ears is 10.5 years by endpoint of moderately severe SNHL, and 7.1 years by endpoint of WRS<60%.

*Time to hearing loss progression in contralateral ears with abnormal ISSNHL-ipsilateral hearing*

Figure S3 shows that patients with recovery to abnormal hearing after ISSNHL, but with normal hearing still in the contralateral ear, were significantly more likely than controls to demonstrate a decline in hearing in the remaining better-hearing ear. This finding was significant by endpoint of moderate hearing loss (p<0.0001), moderately severe hearing loss (p=0.0002), and WRS<78% (p=0.0034), however non-significant for endpoint of WRS<60%. In these ears, median time to reach endpoint of moderate hearing loss was 13.9 years, and to reach endpoint of WRS<78% was 12.8 years.

*Risk factor assessment for hearing loss progression after recovery to normal hearing post-ISSNHL*

Figures S4 and S5 outline the influence of patient factors for reaching hearing loss endpoint at 5 years post-ISSNHL, in patients with normal hearing bilaterally following ISSNHL recovery, in the ISSNHL-ipsilateral ear; Figure 4 evaluates patients by threshold endpoint, while Figure 5 does the same for word understanding endpoint. Regarding threshold endpoints, male sex (OR 1.35, p=0.023), hypertension (OR 2.70, p=0.011) and abnormal contralateral hearing (OR 2.47, p=0.045) were all found to significantly increase risk of achieving moderate hearing loss at 5 years following initial ISSNHL and recovery. For endpoint of moderately severe hearing loss, male sex (OR 1.47, p=0.0072) and abnormal contralateral hearing (OR 2.97, p=0.042) were even more strongly associated with increased risk for hearing loss progression. Regarding word understanding endpoint, male sex (OR 1.45, p=0.0020), hypertension (OR 2.23, p=0.033), diabetes (OR=6.09, p=0.014) and abnormal contralateral hearing (OR 2.32, p=0.039) were all found to significantly increase risk of reaching WRS<78% at 5 years following initial ISSNHL and recovery. For endpoint of WRS<60%, male sex (OR 1.48, p=0.0036) and abnormal contralateral hearing (OR 2.95, p=0.018) were also associated with increased risk for hearing loss progression. Odds ratios were also calculated after weighting male and female populations by underlying patient comorbidities that had been found to be more prevalent in the males as described in Table 1 – hypertension, hyperlipidemia, diabetes, and coronary artery disease. Using this approach, the significance of male sex alone disappeared for all endpoints assessed in Figures S4 and S5.

Figure S6 outlines the influence of patient factors for reaching hearing loss endpoint at 5 years post-ISSNHL, in patients with only partial post-ISSNHL recovery in hearing, in the ISSNHL-ipsilateral ear. In patients with post-ISSNHL recovery to mild hearing loss, male sex (OR 1.49, p=0.037), age <60 years (OR 1.51, p=0.028) and tinnitus (OR 1.50, p=0.0016) were all associated with increased risk of reaching moderately severe hearing loss endpoint at 5 years post-ISSNHL. In patients with post-ISSNHL recovery to WRS 78-<92%, only vertigo was associated with increased risk of reaching WRS<60% endpoint (OR 2.66, p=0.025). Odds ratios were also calculated after weighting male and female populations by underlying patient comorbidities that had been found to be more prevalent in the males as described in Table 1 – hypertension, hyperlipidemia, diabetes, and coronary artery disease. Using this approach, the significance of male sex alone disappeared for all endpoints assessed in Figure SS.

Figures S7 and S8 outline the influence of patient factors for reaching hearing loss endpoint in the ISSNHL-contralateral ear at 5 years post-ISSNHL, in patients with normal hearing in only the contralateral (unaffected) ear following ISSNHL recovery. Figure S7 evaluates patients by threshold endpoint, while Figure S8 does the same for word understanding endpoint. Regarding threshold endpoints, only male sex was found to significantly increase risk of achieving moderate hearing loss at 5 years following initial ISSNHL and recovery (OR 1.57, p=0.0015). Odds ratios were also calculated after weighting male and female populations by underlying patient comorbidities that had been found to be more prevalent in the males as described in Table 1 – hypertension, hyperlipidemia, diabetes, and coronary artery disease. Using this approach, the significance of male sex for progression to moderate hearing loss was reduced, but still maintained significance (OR 1.44, p=0.012). For endpoint of moderately severe hearing loss, none of the patient factors tested were found to be associated with increased risk for hearing loss progression. Regarding word understanding endpoint, age >60 years (OR 1.62, p=0.0013) and abnormal contralateral hearing (OR 1.45, p=0.037) were found to significantly increase risk of reaching WRS<78% at 5 years following initial ISSNHL and recovery. For endpoint of WRS<60%, only vertigo was found to be associated with increased risk for hearing loss progression (OR 4.92, p<0.0001).

**Discussion**

Results of these extended analyses generally confirm the findings from the main body of the manuscript. While univariate analysis consistently shows males to be at higher risk for hearing loss progression post-ISSNHL, controlling for the demographic factors that are more prevalent within the male population for this study reliably relegates this trend to non-significance. Of note, males are actually over-represented in the patient group with recovery to normal hearing bilaterally – this finding could be explained by a greater degree of reversibility in mechanisms that are predominantly common in the male population, such as the cardiovascular risk factors noted above, while other mechanisms that are relatively more prevalent in the female population are less likely to be reversible (6). While similar trends are seen in the bilateral ISSNHL group, with males representing 6/7 patients with bilateral hearing loss and full recovery, this trend is not significant.

**Supplementary Tables**

**Table S1:** Demographics in patients with audiometrically-confirmed unilateral ISSNHL, segmented by degree of hearing recovery post-ISSNHL.

|  | **Normal baseline hearing**  **in ISSNHL-ipsilateral ear**  ***(contralateral hearing normal)*** | | | **Abnormal baseline hearing**  **in ISSNHL-ipsilateral ear**  ***(contralateral hearing normal)*** | | |
| --- | --- | --- | --- | --- | --- | --- |
|  | **PTA ≤25dB** | **WRS ≥92%** | **PTA≤25dB**  ***and***  **WRS≥92%** | **PTA >25dB** | **WRS <92%** | **PTA>25dB**  ***and***  **WRS<92%** |
| **Total N** | 170 | 199 | 150 | 294 | 279 | 234 |
| **Mean Age (years)** | 54.5* | 55.4 | 54.4 | 57.2* | 57.5 | 56.6 |
| **Male N****  **(%)** | 96  (56.5%) | 112  (56.3%) | 82  (54.7%) | 140  (47.6%) | 130  (46.6%) | 107  (45.7%) |
| **Female N****  **(%)** | 74  (43.5%) | 87  (43.7%) | 68  (45.3%) | 154  (52.4%) | 149  (53.4%) | 127  (54.3%) |
| * Significant (p=0.030) difference in age between patients with ISSNHL-ipsilateral normal vs. abnormal hearing by threshold criteria; all other age differences between groups are non-significant.  ** Significant difference in sex distribution between patients with ISSNHL-ipsilateral normal vs. abnormal hearing; p=0.010 by threshold criteria, p=0.0011 by word understanding criteria, and p=0.028 by combined criteria.  Outputs plotted in Figures S1 and S3 are based on most restrictive hearing definitions for normal vs. abnormal baseline hearing, with normal ISSNHL-contralateral hearing in all patients, as outlined in red. | | | | | | |

**Table S2:** Demographics in patients with audiometrically-confirmed unilateral ISSNHL and reduced ipsilateral hearing post-ISSNHL.

|  | **Reduced baseline hearing**  **in ISSNHL-ipsilateral ear**  ***(any contralateral hearing)*** | |
| --- | --- | --- |
|  | **PTA**  **>25-40dB** | **WRS**  **78%-<92%** |
| **Total N** | 114 | 89 |
| **Mean Age (years)** | 57.5 | 57.2 |
| **Male N**  **(%)** | 53  (46.5%) | 47  (52.8%) |
| **Female N**  **(%)** | 61  (53.5%) | 42  (47.2%) |
| Outputs plotted in Figure S2 are derived from patient populations in the above table and are based on post-ISSNHL ipsilateral hearing status only, without controlling for contralateral hearing. | | |

**Figure S1.** Kaplan-Meier curves for hearing loss progression in patients with post-ISSNHL normal hearing (WRS≥92% and PTA≤25dB) bilaterally; see Table S1 for demographics. **A)** Endpoint bone three-tone PTA>40dB (moderate HL); **B)** Endpoint bone three-tone PTA>55dB (moderately severe HL); **C)** Endpoint word understanding <78%; **D)** Endpoint word understanding <60%. All control ears with normal baseline hearing defined by same criteria as for ISSNHL-ipsilateral and contralateral ears.

**Figure S2.** Kaplan-Meier curves for hearing loss progression in patients with post-ISSNHL reduced hearing in the ipsilateral ear, either by PTA>25-40dB in **A**, or by WRS 78-<92% in **B**; see Table S2 for demographics. **A)** Endpoint bone three-tone PTA>55dB (moderately severe HL); **B)** Endpoint word understanding <60%. All control ears with reduced baseline hearing defined by same criteria as for ISSNHL-ipsilateral ears.

**Figure S3.** Kaplan-Meier curves for hearing loss progression in patients with post-ISSNHL abnormal hearing in the ipsilateral ear (WRS<92% and PTA>25dB) and baseline normal hearing in the contralateral ear (WRS≥92% and PTA≤25dB); see Table S1 for demographics. **A)** Endpoint bone three-tone PTA>40dB (moderate HL); **B)** Endpoint bone three-tone PTA>55dB (moderately severe HL); **C)** Endpoint word understanding <78%; **D)** Endpoint word understanding <60%. All control ears with normal baseline hearing defined by same criteria as for ISSNHL-ipsilateral and contralateral ears.

**Figure S4.** Forest plot for odds ratio to reach endpoint by 3-tone PTA criteria in the ISSNHL-ipsilateral ear at 5 years, in patients with recovery to normal ipsilateral thresholds post-ISSNHL.

**Figure S5.** Forest plot for odds ratio to reach endpoint by WRS criteria in the ISSNHL-ipsilateral ear at 5 years, in patients with recovery to normal ipsilateral WRS post-ISSNHL.

**Figure S6.** Forest plot for odds ratio to reach endpoint by either by 3-tone PTA or WRS criteria in the ISSNHL-ipsilateral ear at 5 years, in patients with reduced hearing post-ISSNHL.

**Figure S7.** Forest plot for odds ratio to reach endpoint by 3-tone PTA criteria in the ISSNHL-contralateral ear at 5 years, in patients with normal contralateral thresholds post-ISSNHL.

**Figure S8.** Forest plot for odds ratio to reach endpoint by WRS criteria in the ISSNHL-contralateral ear at 5 years, in patients with normal contralateral WRS post-ISSNHL.

**References**

1. Kim JM, Na MS, Jung KH, Lee SH, Han JS, Lee OH, et al. The Best-Matched Pure Tone Average and Speech Recognition Threshold for Different Audiometric Configurations. Korean J Otorhinolaryngol-Head Neck Surg. 2016;59(10):725-9.

2. Huh DA, Choi YH, Ji MS, Moon KW, Yoon SJ, Sohn JR. Comparison of Pure-Tone Average Methods for Estimation of Hearing Loss Caused by Environmental Exposure to Lead and Cadmium: Does the Pure-Tone Average Method Which Uses Low-Frequency Ranges Underestimate the Actual Hearing Loss Caused by Environmental Lead and Cadmium Exposure? Audiol Neurootol. 2018;23(5):259-69.

3. Early S, Rinnooy Kan CE, Eggink M, Frijns JHM, Stankovic KM. Progression of Contralateral Hearing Loss in Patients With Sporadic Vestibular Schwannoma. Front Neurol. 2020;11:796.

4. Halpin C, Rauch SD. Using audiometric thresholds and word recognition in a treatment study. Otol Neurotol. 2006;27(1):110-6.

5. Snel-Bongers J, Netten AP, Boermans PBM, Rotteveel LJC, Briaire JJ, Frijns JHM. Evidence-Based Inclusion Criteria for Cochlear Implantation in Patients With Postlingual Deafness. Ear Hear. 2018;39(5):1008-14.

6. Xie W, Dai Q, Liu J, Liu Y, Hellström S, Duan M. Analysis of Clinical and Laboratory Findings of Idiopathic Sudden Sensorineural Hearing Loss. Sci Rep. 2020;10(1):6057.
